# Application of Octanohydroxamic Acid for Salting out Liquid–Liquid Extraction of Materials for Energy Storage in Supercapacitors

**DOI:** 10.3390/molecules26020296

**Published:** 2021-01-08

**Authors:** Kaelan Rorabeck, Igor Zhitomirsky

**Affiliations:** Department of Materials Science and Engineering, McMaster University, Hamilton, ON L8S 4L7, Canada; rorabeck@mcmaster.ca

**Keywords:** manganese oxide, supercapacitor, salting-out, dispersion, extraction, capacitance

## Abstract

The ability to achieve high areal capacitance for oxide-based supercapacitor electrodes with high active mass loadings is critical for practical applications. This paper reports the feasibility of the fabrication of Mn_3_O_4_-multiwalled carbon nanotube (MWCNT) composites by the new salting-out method, which allows direct particle transfer from an aqueous synthesis medium to a 2-propanol suspension for the fabrication of advanced Mn_3_O_4_-MWCNT electrodes for supercapacitors. The electrodes show enhanced capacitive performance at high active mass loading due to reduced particle agglomeration and enhanced mixing of the Mn_3_O_4_ particles and conductive MWCNT additives. The strategy is based on the multifunctional properties of octanohydroxamic acid, which is used as a capping and dispersing agent for Mn_3_O_4_ synthesis and an extractor for particle transfer to the electrode processing medium. Electrochemical studies show that high areal capacitance is achieved at low electrode resistance. The electrodes with an active mass of 40.1 mg cm^−2^ show a capacitance of 4.3 F cm^−2^ at a scan rate of 2 mV s^−1^. Electron microscopy studies reveal changes in electrode microstructure during charge-discharge cycling, which can explain the increase in capacitance. The salting-out method is promising for the development of advanced nanocomposites for energy storage in supercapacitors.

## 1. Introduction

Supercapacitors are currently under development for various energy storage applications [1,2]. Investigations in this research area are focused on the synthesis of advanced electrode materials by different fabrication methods [3,4,5,6,7,8], optimization of electrolytes [9], design of nanocomposites [10,11,12,13], and modeling [14]. The synthesis of non-agglomerated particles of controlled shape and composition is of particular importance for supercapacitor technology [15,16,17]. Many investigations have focused on the manufacturing of wearable and flexible devices [18,19,20]. There has been significant progress in the development of advanced electrode microstructures [21,22,23], which facilitate charge-discharge reactions.

Recent studies have highlighted the need for the development of efficient electrodes with high active mass loading and the low mass of passive components [24]. It should be noted that high gravimetric capacitance is achieved only in thin films. However, the increase in active mass results in poor electrolyte access to the active material and high resistance. Therefore, the gravimetric capacitance decreases with increasing active mass. Improved material performance at high active mass is achieved using advanced techniques for the synthesis of porous materials and the design of advanced structures by template methods [24,25,26]. Novel research avenues have been proposed for the fabrication of nanoparticles of controlled size and shape [27,28]. The capacitive properties of electrodes are enhanced by the development of nanorods, nanowires, nanotubes, as well as lamellar, hollow, and core-shell particles of different capacitive materials [27,29,30]. New strategies have been developed for the fabrication of nanocomposites by heterocoagulation techniques, which involve electrostatic interactions or Schiff base reactions [24]. Significant progress has been achieved in the development of new dispersing agents for inorganic particles, graphene, carbon nanotubes, and other capacitive materials and conductive additives [31,32].

The need for supercapacitor electrodes with enhanced performance at high active mass has driven the development of particle extraction through a liquid–liquid interface (PELLI) method [33] for agglomerate-free processing of inorganic nanoparticles. The method allows direct particle transfer from the synthesis medium to a device processing medium, avoiding particle drying and re-dispersion stages. It has been shown [33] that the driving forces for particle agglomeration during the drying stage are the reduction of surface area and surface condensation reactions. Different PELLI strategies have been developed, such as bottom-up and top-down strategies. Various chelating organic molecules have been utilized as extractors, enabling particle transfer through the interface of two immiscible liquids [33]. The PELLI method has allowed for enhanced mixing of non-agglomerated particles of different capacitive materials with conductive additives and fabrication of electrodes with high areal capacitance and low resistance [24], which are achieved as a result of an enhanced material performance at high active mass. Despite the success in the application of PELLI in the development of advanced electrodes with high areal capacitance, the PELLI technique has limitations. Particle synthesis is usually achieved in an aqueous phase, and it is challenging to avoid agglomeration during synthesis. Extractor molecules are usually accumulated at the liquid–liquid interface with their hydrophilic groups exposed to water and hydrophobic hydrocarbon chains extending into the organic phase. Inorganic particles are modified at liquid–liquid extraction (LLI) and extracted into the organic phase after synthesis. Hydrophobic receiving liquids, such as 1-butanol, are immiscible with water. Moreover, 1-butanol has a relatively high boiling point. As a result, it is difficult to remove adsorbed 1-butanol molecules from the particle surface. The adsorbed hydrophobic 1-butanol molecules are detrimental to the access of an aqueous electrolyte to the particle surface.

The goal of this investigation was to develop Mn_3_O_4_-multiwalled carbon nanotube (MWCNT) electrodes using a new extraction strategy and avoid the limitations of the PELLI method. The approach was based on the use of 2-propanol as a receiving liquid instead of 1-butanol. It is important to note that 2-propanol is miscible with water. However, 2-propanol can be separated from an aqueous phase by the addition of salts, such as NaCl. The approach developed in this investigation involved the synthesis of Mn_3_O_4_ in an aqueous phase, the addition of a suspension of MWCNT in 2-propanol, co-dispersion, and mixing of Mn_3_O_4_ in the water-2-propanol mixture, salting out the 2-propanol phase, and extraction of Mn_3_O_4_ and MWCNT into the 2-propanol phase. The approach is conceptually different from the partitioning of organic compounds in a mixture of water and an organic solvent using a salting-out effect. In our investigation, Mn_3_O_4_ particles were synthesized in an aqueous phase. The synthesis was performed in the presence of an extractor, which acted as a capping agent for the synthesis and a dispersant for Mn_3_O_4_. The phase separation of aqueous and 2-propanol phases was achieved after synthesis by the addition of NaCl. The method allowed for the extraction of Mn_3_O_4_ and carbon nanotubes into the 2-propanol phase and their efficient dispersion and mixing. In this approach, the problem related to the adsorption of hydrophobic 1-butanol on the particle surface was avoided. The use of octanohydroxamic acid (OHA) as an extractor, capping, and the dispersing agent was a key for the successful fabrication of high active mass electrodes with high areal capacitance and low impedance by the salting-out particle extraction.

## 2. Results and Discussion

**Figure 1:**
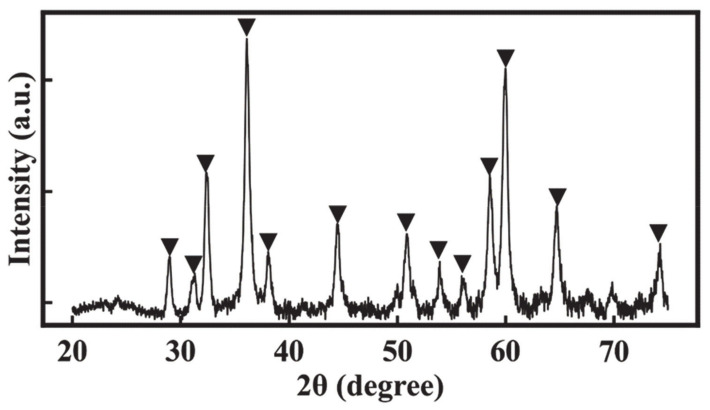
X-ray diffraction pattern of the as precipitated material (▼—peaks corresponding to JCPDS file 24-0734 of Mn_3_O_4_).

In this investigation, crystalline Mn_3_O_4_ material was prepared by a chemical precipitation method. Figure 1 shows the X-ray diffraction pattern of the obtained material. It exhibits diffraction peaks, corresponding to the JCPDS file 24-0734 of Mn_3_O_4_. For the fabrication of supercapacitor electrodes, Mn_3_O_4_ particles must be transferred from the aqueous synthesis media to an organic solvent containing a dissolved binder. A water-insoluble binder provides electrode stability in an aqueous electrolyte. Mn_3_O_4_ particles must be co-dispersed with conductive MWCNT in an organic solvent to achieve good mixing.

**Figure 2:**
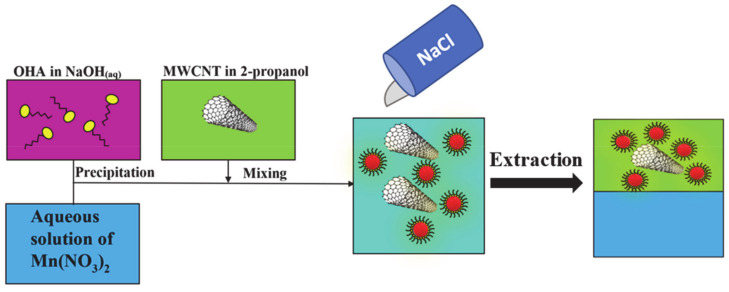
Schematic of Mn_3_O_4_ synthesis, mixing with multiwalled carbon nanotube (MWCNT), and salting-out method.

**Figure 3:**
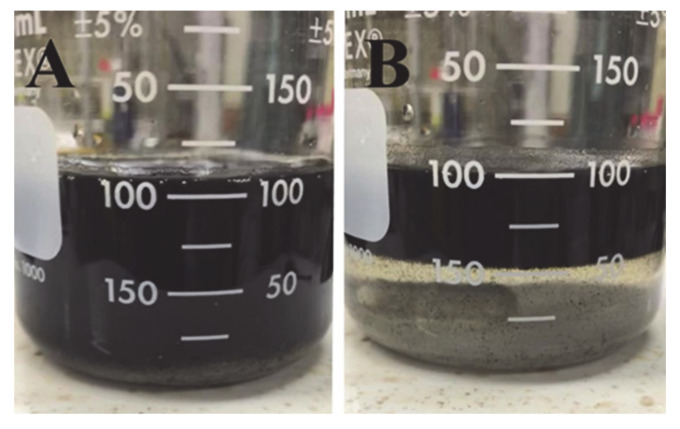
Suspension of Mn_3_O_4_ and MWCNT dispersed using octanohydroxamic acid (OHA) in a mixed water-2-propanol solvent (**A**) before and (**B**) after adding NaCl.

A traditional strategy involves washing, drying, and redispersion of particles in an organic solvent. However, as pointed out above, drying leads to particle agglomeration, which results in their poor mixing with conductive additives and poor electrode performance. PELLI method [33] allows for the direct transfer of oxide particles from an aqueous synthesis medium to an organic medium for electrode fabrication, thus avoiding particle agglomeration during the drying step. In many previous investigations, 1-butanol was used as a receiving liquid, which is immiscible with water. The superior capacitive behavior of electrodes, prepared using PELLI [33], was linked to the reduced agglomeration of capacitive materials and their improved mixing with conductive additives. However, there are difficulties related to the adsorption of hydrophobic 1-butanol on the surface of inorganic particles and carbon nanotube additives. The adsorbed hydrophobic 1-butanol limits the access of aqueous electrolyte to the active material surface. The relatively high boiling point of 1-butanol introduces problems with its removal. This reduces the benefits of PELLI for the fabrication of supercapacitor electrodes. To overcome these obstacles, we explored the salting-out method, which is conceptually different from the PELLI method. Figure 2 shows a schematic of the procedure used for Mn_3_O_4_ synthesis, mixing with MWCNT, and salting out of the mixture of Mn_3_O_4_ and MWCNT to the 2-propanol phase. Figure 3 shows a suspension of Mn_3_O_4_ and MWCNT dispersed using OHA in a mixture of water and 2-propanol before and after salting out.

The salting-out method does not involve particle transfer through the liquid–liquid interface. It is based on the use of 2-propanol as a receiving liquid, which is miscible with water and has a relatively low boiling point. Moreover, 2-propanol and water form an azeotrope at a 2-propanol concentration of 87 wt% with a low boiling point of 80.3 °C [34]. The salting-out method was used for the separation of 2-propanol from an azeotropic water-2-propanol mixture [34]. It has previously been shown that salting out phase separation of 2-propanol and water can be used for the selective extraction of Co(II) [35] and Au(III) [36] ions. In our investigation, the salting-out method was developed for the extraction of Mn_3_O_4_ particles and MWCNT. In this approach, salting out resulted in a separation of 2-propanol, containing Mn_3_O_4_ and MWCNT from the aqueous phase. The co-extraction of Mn_3_O_4_ and MWCNT to 2-propanol allowed the fabrication of a composite electrode by impregnation of a current collector using a suspension of Mn_3_O_4_ and MWCNT.

**Figure 4:**
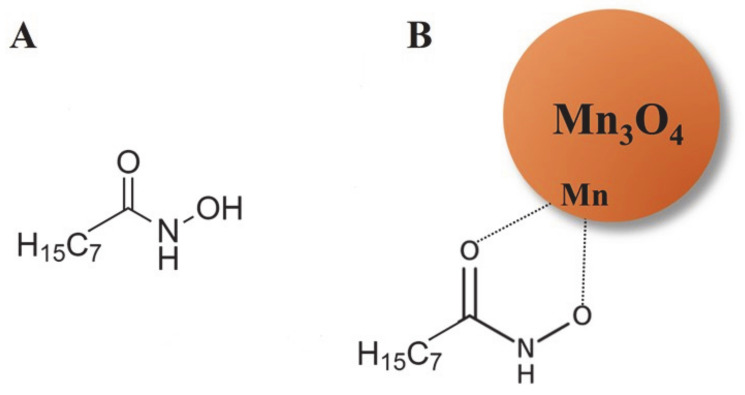
(**A**) The chemical structure of OHA, (**B**) OHA adsorption on Mn_3_O_4_ particles, involving complexation of surface Mn atoms.

In experiments performed without OHA, the hydrophobic MWCNT was extracted to the 2-propanol phase, whereas Mn_3_O_4_ remained in the aqueous phase. The modification of Mn_3_O_4_ particles with OHA was necessary for their extraction. Figure 4A shows a schematic of the chemical structure of OHA, which contains an alkyl chain, carbonyl, and NOH_2_ groups. It has previously been shown that hydroxamic acids and their derivatives, such as OHA, exhibit metal chelating properties, which involve interactions of carbonyl and NOH_2_ groups of the molecules with metal atoms, and facilitate their adsorption on surfaces of metal oxides [37,38,39,40]. Figure 4B shows a suggested mechanism of OHA adsorption on Mn_3_O_4_ particles, which involves the complexation of Mn atoms on the particle surface. In this investigation, OHA was used as a capping and dispersing agent for Mn_3_O_4_ synthesis. The hydrophobic alkyl chain of the adsorbed OHA molecule facilitated particle transfer into the 2-propanol phase. The transfer of Mn_3_O_4_ and MWCNT into the 2-propanol phase facilitated their co-dispersion and mixing, which allowed for the fabrication of electrodes with high areal capacitance at high active mass loading. The composite electrode was a mixture of Mn_3_O_4_ and MWCNT.

**Figure 5:**
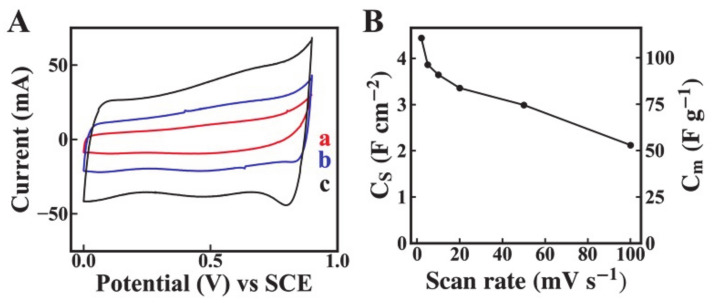
(**A**) CVs at scan rates of (a) 2, (b) 5, (c) 10 mV s^−1^, and (**B**) C_S_ and C_m_ versus scan rate for Mn_3_O_4_-MWCNT electrodes.

Figure 5A shows Cyclic voltammetry (CV) curves for Mn_3_O_4_-MWCNT electrode with an active mass of 40.1 mg cm^−2^ prepared using the salting-out method. The nearly rectangular shape of the CVs and increase in the CV area with increasing scan rate indicate good capacitive behavior. The highest capacitance of 4.3 F cm^−2^ was achieved (Figure 5B) at a scan rate of 2 mV s^−1^. The obtained capacitance was higher than literature data for Mn_3_O_4_-MWCNT electrodes, prepared by different advanced methods and summarized in a recent review [24]. The electrodes showed a high capacitance of 2.1 F cm^−2^ at a scan rate of 100 mV s^−1^ with capacitance retention of 48.8%. The obtained areal capacitance was higher than that obtained in other investigations of electrodes with high active mass [41,42,43]. It is important to note that MWCNT was used as an additive, which enhanced the electrical conductivity of the composite electrodes. It was found that the capacitance of pure MWCNT electrodes with a mass of 8 mg cm^−2^ was 0.032 F cm^−2^ at a scan rate of 2 mV s^−1^. Therefore, the contribution of the capacitance of MWCNT to the total capacitance was below 1%.

**Figure 6:**
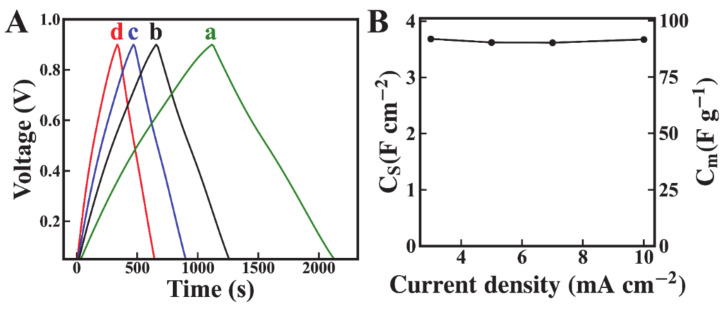
(**A**) Charge discharge behavior at current densities of (a) 3, (b) 5, (c) 7, and (d) 10 mA cm^−2^, and (**B**) capacitance versus current density for Mn_3_O_4_-MWCNT electrodes.

Figure 6 shows charge-discharge data for the Mn_3_O_4_-MWCNT electrodes at different current densities. The time dependences of charge and discharge currents were nearly linear, and the charge-discharge curves were of a symmetric triangular shape. The capacitance C_S_ calculated at a discharge current of 3 mA cm^−2^ was 3.8 F cm^−2^. It was found that C_s_ was nearly constant at discharge currents of 3–10 mA cm^−2^.

**Figure 7:**
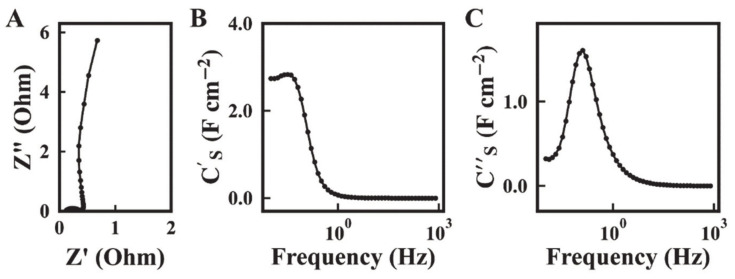
(**A**) Nyquist plot of complex impedance and (**B**,**C**) frequency dependences of (**B**) C_S_′ (**C**) C_S_″ for Mn_3_O_4_-MWCNT electrodes.

The Nyquist plot of complex impedance showed a nearly vertical line, which indicated good capacitive behavior (Figure 7A). The low real part of impedance resulted from good mixing of Mn_3_O_4_ with conductive MWCNT. The electrode resistance, represented by the real part of the complex impedance, decreased with increasing frequency. The highest resistance of 0.68 Ohm was obtained at a frequency of 10 mHz.

**Figure 8:**
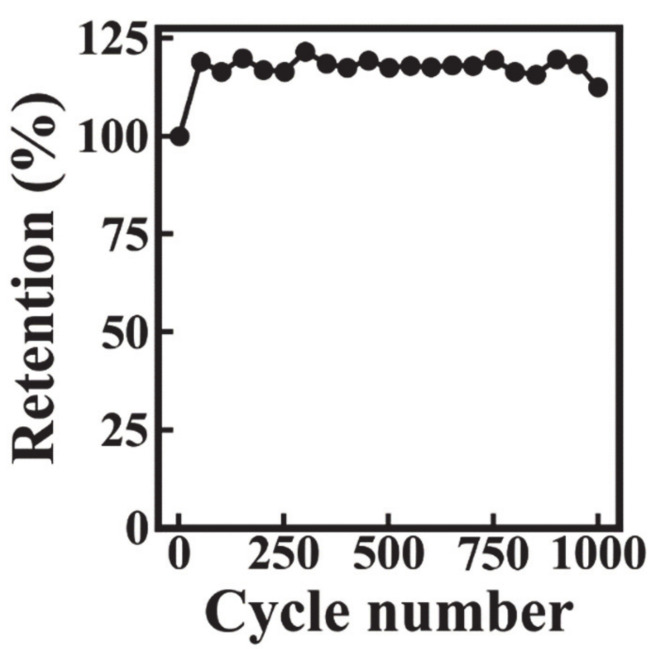
Capacitance retention versus cycle number for Mn_3_O_4_-MWCNT electrodes.

**Figure 9:**
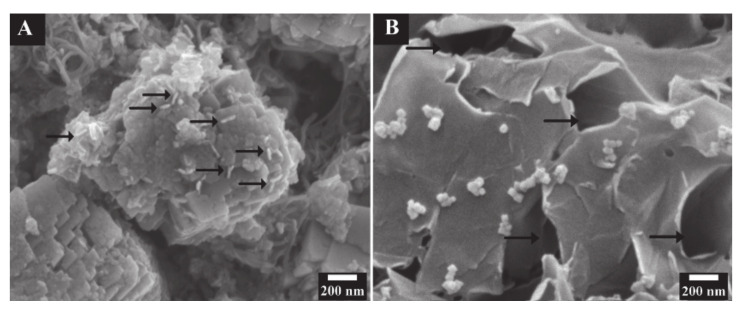
SEM images of the Mn_3_O_4_-MWCNT electrodes: (**A**) as prepared, arrows show MWCNT in the Mn_3_O_4_ matrix, (**B**) after cycling, arrows show large pores.

The capacitance C_S_′ obtained at AC voltage of 5 mV (Figure 7B) was lower than C_S_ obtained from the CV data in a voltage window of 0.9 V. The difference can be attributed to limited electrolyte ion access to the active material at low voltages. The capacitance C_S_′ decreased with increasing frequency, and the frequency dependence of C_S_″ showed a maximum (Figure 7C), which is typical for a relaxation type dispersion [15]. Figure 8 shows the cyclic behavior of the Mn_3_O_4_-MWCNT electrode. The capacitance showed an initial increase during the first 50 cycles and then remained nearly constant. The capacitance retention after 1000 cycles was 118%. It was hypothesized that the increase of capacitance during cycling could be attributed to the changing of the wetting behavior of the active material by the electrolyte. Figure 9 shows SEM images of the electrodes before and after cycling. The SEM image of the electrode before cycling showed composite particles with a typical size of 1–2 μm. The composite particles contained MWCNT dispersed in the Mn_3_O_4_ matrix. Such microstructure results from good mixing of the Mn_3_O_4_ particles and MWCNT in the suspension, prepared by the salting-out method for impregnation of current collectors, and explains the good capacitive behavior and low impedance of the electrodes. The SEM image of the electrodes after cycling shows a flaky morphology and large pores. It is suggested that such pores facilitated electrolyte access to the bulk of the electrodes and could be considered as an additional factor, which resulted in increased capacitance during cycling.

## 3. Materials and Methods

Octanohydroxamic acid (TCI America), Mn(NO_3_)_2_^.^4H_2_O, NaOH, Na_2_SO_4_, 2-propanol, NaCl, polyvinylbutyral (PVB) (Aldrich, Oakville, ON, Canada), multiwalled carbon nanotubes (Bayer, Germany) were used as starting materials. The 330 mg of Mn(NO_3_)_2_∙4H_2_O was dissolved into 20 mL of DI water, and then the pH was adjusted to 10 by the addition of aqueous NaOH solution, containing 33 mg of dissolved OHA. Obtained suspension of Mn_3_O_4_ particles modified with OHA as a capping and dispersing agent was ultrasonicated, and then 25 mg of MWCNT dispersed in 30 mL of 2-propanol was added. OHA improved dispersion of MWCNT, and a stable suspension was obtained. The addition of 6 g NaCl resulted in the separation of aqueous and 2-propanol phases. The phase separation resulted in a transfer of Mn_3_O_4_ and MWCNT into the 2-propanol phase (Figure 1 and Figure 2) to form a composite suspension, containing Mn_3_O_4_ and MWCNT in a mass ratio of 4:1. The suspension of Mn_3_O_4_ and MWCNT in 2-propanol was separated from the aqueous phase, and the PVB binder was added to the suspension in order to achieve a ratio of PVB/(Mn_3_O_4_ + MWCNT) = 0.03.

The suspension was used for the impregnation of commercial Ni foam (Vale, Toronto, ON, Canada) current collectors. The mass ratio of PVB:Mn_3_O_4_:MWCNT in the electrode was 3:80:20. Due to the low boiling point of 2-propanol, it was removed from the electrode material surface by drying of the electrode at 60 °C. The electrode area was 1 cm^2^. The total mass of the material (active mass) impregnated into the current collector was 40.1 (±0.03) mg cm^−2^. The electrode thickness was 0.4 mm.

X-ray diffraction (XRD) analysis (diffractometer Bruker D8, UK) was performed using Cu-Kα radiation in the 2θ range of 8–135 degrees, at the rate of 0.01 degrees per second. Electrode microstructures were analyzed using a JEOL SEM (scanning electron microscope, JSM-7000F) at an applied voltage of 5 keV and a working distance of 6 mm. Cyclic voltammetry (CV) and electrochemical impedance spectroscopy (EIS) studies were performed using a potentiostat-impedance analyzer PARSTAT 2273 (Ametek). Cyclic voltammetry data was obtained at sweep rates of 2–100 mV s^−1^. EIS data was obtained at an open circuit potential using alternating voltage with an amplitude of 5 mV in the frequency range of 0.01–10 kHz. Galvanostatic charge-discharge (GCD) investigations in a fixed potential range were performed using BioLogic VMP 300 at current densities of 3, 5, 7, and 10 mA cm^−2^. Testing was performed using a 3-electrode electrochemical cell containing a working electrode (impregnated Ni foam), counter-electrode (Pt mesh), and a reference electrode (SCE, saturated calomel electrode). Aqueous 0.5 M Na_2_SO_4_ solution was used as an electrolyte. Three different methods were used for the calculation of capacitance.

The integral capacitance C in the voltage window 0–0.9 V versus SCE was calculated from the CV data

(1) $$C=\frac{\Delta Q}{\Delta U}=\frac{|\int_{0}^{t\left(\mathrm{Umax}\right)}\mathrm{Idt}|+|\int_{t\left(\mathrm{Umax}\right)}^{0}\mathrm{Idt}|\;}{2\mathrm{Umax}}$$

where ΔQ denotes charge, I—current, t—time, and ΔU—the potential range.

The integral capacitance was also calculated from the GCD data in the same potential range:

(2) C =IΔt/ΔU

where I denotes the applied current, and Δt—discharge time.

The differential complex capacitance C*(ω) = C′(ω) − Ic″(ω) was calculated at different frequencies (ω) from the complex impedance Z*(ω) = Z′(ω) + *i* Z″(ω) data [44], where

(3) $$C\prime \left(\omega \right)\;=\frac{-Z\prime \prime \left(\omega \right)}{\omega |Z{\left(\omega \right)|}^{2}}$$

(4) $$C\prime \prime \left(\omega \right)\;=\frac{Z\prime \left(\omega \right)}{\omega |Z{\left(\omega \right)|}^{2}}$$

The capacitive properties of electrode material were presented in gravimetric (C_m_) and areal (C_S_) capacitance forms.

## 4. Conclusions

For the first time, the salting-out method was used for the extraction of particles from the aqueous synthesis medium to an electrode processing organic medium. OHA was used as a capping and dispersing agent for the synthesis as well as a particle extractor. The adsorption of OHA on Mn_3_O_4_ particles involved the complexation of Mn atoms on the particle surface. The surface modification of Mn_3_O_4_ particles with OHA facilitated the particle transfer into the 2-propanol phase. The method eliminated the particle drying stage, which resulted in particle agglomeration. The method facilitated co-dispersion of Mn_3_O_4_ and MWCNT in the 2-propanol phase and their enhanced mixing, which allowed for the fabrication of high active mass electrodes with enhanced performance. A capacitance of 4.3 F cm^−2^ was achieved at low impedance at active mass loading of 40.1 mg cm^−2^. The electrodes showed good capacitance retention at high scan rates and good cyclic stability. The initial capacitance increase during cycling could be attributed to the changes in microstructure. The method opens a new and unexplored path for the fabrication of nanocomposites. Further progress in applications of the salting-out method will depend on the development of advanced extractor molecules.

## Data Availability

The data presented in this study are available in: Application of Octanohydroxamic Acid for Salting out Liquid–Liquid Extraction of Materials for Energy Storage in Supercapacitors.
